# A Curious Case of Diabetic Ketoacidosis Secondary to Avelumab

**DOI:** 10.7759/cureus.62240

**Published:** 2024-06-12

**Authors:** Mafalda Costa, Helena Magalhães

**Affiliations:** 1 Department of Medical Oncology, Pedro Hispano Hospital, Matosinhos, PRT

**Keywords:** avelumab, urothelial cancer, diabetic keto-acidosis, immune check-point inhibitor, immune mediated side effects

## Abstract

Immune checkpoint inhibitors (ICIs) have completely changed cancer treatment in the last decade and are now widely used in several cancers. In the era of immunotherapy, oncologists have changed not only the way they evaluate treatment efficacy but also the management of treatment-related adverse events. This new profile of immune adverse events has resulted in an urgent need for a more holistic view of cancer patients and for more collaborations with other organ specialists to optimize patient treatment and support. The anti-programmed death-ligand 1 antibody, avelumab, has been widely used as a maintenance treatment in stage IV urothelial carcinoma since the results from the Javelin 100 bladder trial were published. We report a case of a 75-year-old man with stage IV urothelial carcinoma submitted to first-line platinum-based chemotherapy followed by maintenance avelumab. He achieved a complete bone and pulmonary response 10 months after stopping avelumab, which was suspended due to a serious immune adverse event, an ICI-induced type 1 diabetes mellitus. At present, the patient has an overall survival of 24 months and shows no evidence of disease with a good quality of life 16 months after avelumab suspension. We hypothesized that a late response to avelumab could explain this unexpected outcome.

## Introduction

Immune checkpoint inhibitors (ICIs) have a different profile of toxicities known as immune-related adverse events (IRAEs) due to their particular mechanism of action. These agents target immune checkpoints expressed in T cells (CTLA 4 and PD-1) and tumor cells (PDL-1), thereby enhancing cytotoxic T-cell activity and antitumor immune responses [1,2]. However, they may also interfere with immune surveillance, affecting healthy organs and causing a wide spectrum of IRAEs.

The management of these IRAEs requires rapid and efficient multidisciplinary work and collaboration between oncologists and other organ-specific specialists. Several endocrine IRAEs, such as hypophysitis and thyroiditis, have been reported in ICI clinical trials, but new-onset insulin-dependent diabetes is very rare and reported in less than 1% of patients in PD-1 inhibitor clinical trials [4,5]. This condition has not been well addressed in everyday clinical practice, and information is lacking regarding its time of onset, clinical course, and management. With this case report, we intend to share our experience regarding a case of a 75-year-old man with diabetic ketoacidosis secondary to avelumab who presented with a late complete response to treatment for his metastatic urothelial cancer.

## Case presentation

A 75-year-old man with a smoking habit (88 packs per year) and multiple cardiovascular risk factors was evaluated at a urology appointment due to renal lithiasis and a single episode of macroscopic hematuria. Urine cytology revealed atypical urothelial cells and cystoscopy was not well tolerated and inconclusive. At that time, the patient refused transurethral resection of the bladder. Four months later, the patient was admitted to the emergency department with major macroscopic hematuria. A bladder ultrasound revealed a suspicious vegetating lesion 25 mm × 15 mm in size in the left anterolateral wall. A transurethral resection of the bladder was performed, which confirmed the diagnosis of high-grade muscle-invasive urothelial carcinoma. A thoracoabdominal computed tomography (CT) scan showed a lytic bone lesion 26 cm × 15 mm in size in the left ischium, which a biopsy confirmed to be a metastasis of the urothelial carcinoma. The case was discussed by a multidisciplinary team board, and first-line palliative chemotherapy was proposed to the patient.

The patient started first-line chemotherapy with carboplatin 5 AUC and gemcitabine 750 mg/m^2^ with primary prophylaxis for neutropenia. Simultaneously, antalgic radiotherapy to the bone lesion was performed at a total dose of 20 GY/5 fr with three-dimensional conformal radiation therapy (3DCRT). The patient completed six cycles of chemotherapy, and evaluation of the response revealed stable disease.

After multidisciplinary team discussion, maintenance avelumab (800 mg) every two weeks was started. Two days after the second avelumab administration, the patient developed anorexia and prostration, as well as subacute changes in behavior in the previous days. Ten days after cycle 2 administration, he started vomiting repeatedly. For this reason, he was admitted to the emergency room, where he showed impaired mental status, agitation, and changes in his respiratory pattern. At his initial evaluation, the patient was obnubilated, with sparse speech, but he showed no neurological deficits or hemodynamic instability, apart from a respiratory rate of 28 cycles per minute with a Kussmaul respiratory pattern, and normal peripheral oxygen saturation. A rapid glucose test presented extremely high glucose levels (>685 mg/dL) with a ketonemia of 5.8 mmol/L, as confirmed by blood analysis (glucose 998 mg/dL). A brain CT revealed no traumatic, ischemic, or hemorrhagic lesions, but an extensive blood test workup showed a severe acute renal lesion (creatinine of 3.6 mg/dL for a baseline of 0.8 mg/dL) with hyponatremia and hyperkalemia, as well as severe metabolic acidemia (pH < 7), with unmeasurable blood bicarbonate and hyperlactatemia (2.2 mmol/L) (Tables 1-2).

**Table 1:** Laboratory findings at admission (part 1). pO_2_, partial pressure of oxygen; pCO_2_, partial pressure of carbon dioxide; Lact, lactate; Gluc, glucose; Na, sodium; K, potassium

Arterial blood gas analysis (FiO_2_ 21%)	Value	Reference range
pH	<7	7.35-7.45
pO_2_	148 mmHg	75-100 mmHg
pCO_2_	11 mmHg	35-45 mmHg
HCO_3-_	Unmeasurable	22-26 mEq/L
Lact	2.2 mmol/L	0.5-2.0 mmol/L
Gluc	685 mg/dL	70-179 mg/dL
Na^+^	126 mmol/L	133-146 mmol/L
K^+^	5.8 mmol/L	3.5-5.3 mmol/L
Urine analysis		
pH	5.5	4.5-8
Glucose	1000	≤130 mg/dL
Ketone	10	None
Nitrites	Negative	None
Leukocyte	6	<2 to 5
Hemoglobin	500	<5
Cocaine	Negative	None
Cannabinoid	Negative	None
Benzodiazepine	Negative	None
Opioid	Negative	None

**Table 2:** Laboratory findings at admission (part 2). Na, sodium; K, potassium; Fosf, phosphorus; Mg, magnesium; Gluc, glucose; CK, creatine kinase; AST, aspartate aminotransferase; ALT, alanine aminotransferase; T-bil, total bilirubin; WBC, white blood cell count; PLT, platelet count; CRP, C-reactive protein

Blood workup	Value	Reference range
Creatinine	3.6 mg/dL	0.7-1.3 mg/dL
Urea	117 mg/dL	18-55 mg/dL
Na^+^	127 mEq/L	136-145 mEq/L
K^+^	5.7 mEq/L	3.4-5.1 mEq/L
Fosf	8.4 mg/dL	2.3-4.7 mg/dL
Mg	2.88 mg/dL	1.6-2.6 mg/dL
Gluc	998 mg/dL	83-110 mg/dL
CK	700 U/L	30-200 U/L
AST	45 U/L	5-34 U/L
ALT	41 U/L	<55 U/L
T-bil	0.9 mg/dL	0.2-1.2 mg/dL
Hemoglobin	13.5 g/dL	13-18 g/dL
WBC	20,180/µL	4-11,000/µL
PLT	156,000/µL	150-400,000/µL
CRP	45.40 mg/L	<5 mg/L

The patient was started on fluid resuscitation and intravenous insulin and was admitted to the intermediate care unit for close monitoring and study of this new-onset diabetic ketoacidosis and subacute encephalopathy in a patient, with no previous history of diabetes. An endocrinology specialist conducted an extensive workup that revealed HbA1C of 9.4%, low C-peptide, and positive glutamic acid decarboxylase antibody (GAD IgG) (Table 3). The patient had no family history of autoimmune disease. The workup revealed no evidence of adrenal insufficiency or abnormal thyroid function.

**Table 3:** Endocrinology study. HbA1C, hemoglobin A1C; T4L, thyroxine; TSH, thyroid-stimulating hormone

Endocrinology study	Value	Reference range
C-peptide	0.10 ng/mL	0.78-5.19 ng/mL
HbA1C	9.4%	4%-5.9%
Glutamic acid decarboxylase antibody (GAD IgG)	>250 UI/mL	≤10 UI/mL
Anti-zinc transporter protein 8 autoantibodies (ZnT8)	<10	<15
Islet cell cytoplasmic autoantibodies (ICA)	Negative	<1/10 = Negative
Insulinoma-associated-2 autoantibodies (IA2)	<10 UI/mL	≤10 UI/mL
T4L	1.06 ng/dL	0.70-1.48 ng/dL
TSH	0.284 µUI/mL	0.35-4.94 µUI/mL
Anti-TPO antibodies	0.80 UI/mL	<5.61 UI/mL
Cortisol	17.7 µg/dL	3.7-19.4 µg/dL

After multidisciplinary discussion and exclusion of other precipitating factors, the team concluded this to be a case of diabetic ketoacidosis secondary to avelumab that resulted in subacute metabolic encephalopathy. The patient recovered well and was discharged after 15 days with support from endocrinology, along with a prescription for long-acting insulin in the morning and three prandial doses of rapid-acting insulin.

This serious (grade 4) adverse event led to the permanent discontinuation of avelumab. An evaluation CT revealed stable bone disease and new suspicious pulmonary micronodules with a maximum size of 7 mm at multiple locations. The patient was asymptomatic, and surveillance was maintained. Three months later, a new CT scan revealed the progression of the disease and a greater number of micronodules of larger dimensions (10 mm). A next-generation sequencing of the bone metastasis was performed, but no alteration of fibroblast growth factor receptor (FGFR) was found. Treatment with enfortumab vedotin was proposed, but the patient was very reluctant and asked for a treatment holiday.

**Figure 1:**
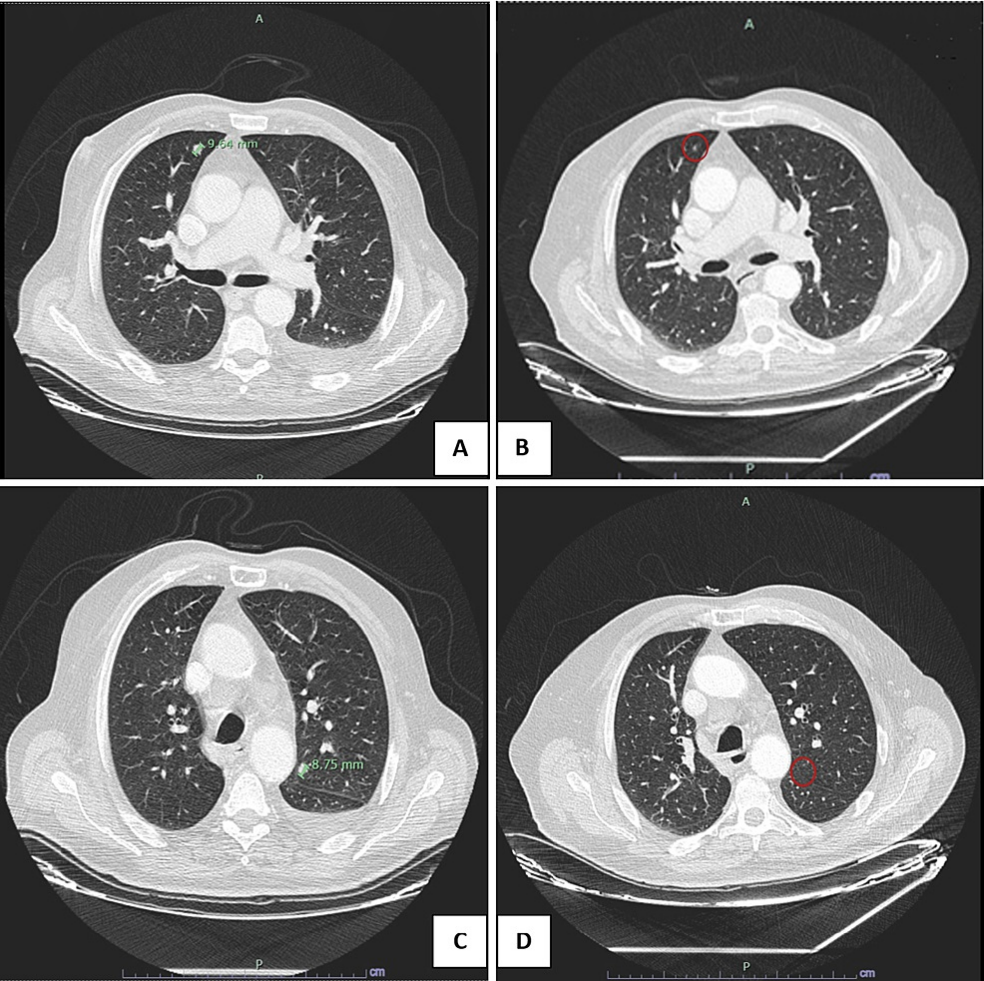
Thoracic CT (A and C) at avelumab discontinuation and (B and D) 10 months after discontinuation. (A and C) Multiple micronodules were visible at various locations with a maximum size of 8-9 mm when avelumab was discontinued. (B and D) The complete response was observed at 10 months with the disappearance of previously described micronodules.

Five months later, the patient started treatment with enfortumab vedotin (1.25 mg/kg). A baseline CT scan revealed a reduction in the pulmonary nodules, which now had a maximum size of 8 mm. However, on day 15 of the first cycle, he presented with grade-three hyperglycemia and poor metabolic control (HbA1C> 8%); therefore, treatment was delayed. After a discussion with the team, the patient decided to discontinue treatment due to poor metabolic control and opted for surveillance instead. Two months later, at 10 months after discontinuation, a new CT revealed a complete response to treatment, which was probably a late response to avelumab (Figure 1). Currently, the patient is insulin-dependent, with reasonable metabolic control, and remains asymptomatic with a good quality of life. He has shown no evidence of disease 16 months after avelumab suspension.

## Discussion

This report presents a case of a 75-year-old male patient with stage IV urothelial carcinoma and a late complete response to avelumab after a serious immune-related adverse event. ICIs have an important role in the treatment landscape of several advanced cancers, and urothelial carcinoma is no exception. Since the results of the Javelin 100 bladder trial, avelumab (anti-PDL1) as a maintenance treatment has become part of the treatment armamentarium for urothelial carcinoma, with an important impact on overall survival upon response to first-line palliative chemotherapy [6]. According to the clinical phase III Javelin 100 bladder trial, only three patients presented with grade three hyperglycemia (0.9%), and no cases of new-onset type 1 diabetes were reported [6].

A published series has shown that in most cases, new-onset diabetes presents at a medium of five months after starting ICI treatment [7]. A high percentage of these patients manifest with diabetic ketoacidosis with low or undetectable C-peptide levels at diagnosis, which reflects the rapid progression of these patients to a severe insulin deficiency state [7]. The mechanism underlying the pathogenesis of ICI-induced diabetes is believed to be an inhibition of the PD-1/PDL-1 pathway and destruction of pancreatic β-cells by activated T cells [8].

Our patient presented with a rapid progression to diabetic ketoacidosis after only two cycles of avelumab treatment. For this reason, clinicians must be aware of this potential life-threatening IRAE and its possible rapid onset. International guideline recommendations also suggest regular monitoring of glucose levels and/or HbA1c during treatment to detect any abnormality as soon as possible [9,10].

In addition to this rapid progression to diabetic ketoacidosis, ICI-induced diabetes shares other similarities with spontaneous type 1 diabetes, as some patients are also positive for autoantibodies of type 1 diabetes, such as glutamic acid decarboxylase antibody (GAD IgG), which was the case for this patient. These ICI-induced diabetes patients also require complex insulin therapy with no possibility of de-escalating insulin treatment, as remission of ICI-induced diabetes is very unlikely [11]. Our patient maintains long-acting insulin and three prandial doses of rapid-acting insulin, but despite all the support from the endocrinology team, he has only reasonable metabolic control and needs a high dose of long-acting insulin. Further knowledge about risk factors for ICI-induced diabetes may improve both the diagnosis and management of this condition and reduce its potential life-threatening and long-term consequences.

Another question raised by this clinical case was the possible association between IRAEs and a better response to ICI treatment. Some published studies and metanalyses have reported better ICI efficacy in patients who experienced IRAEs, particularly adverse endocrine and dermatologic events [5,12-14]. We still know relatively little about predictors of ICI efficacy and toxicity. The rationale for this better response may reflect the fact that an IRAE translates into an uncontrolled immune activation that attacks tumor cells as well as healthy tissue. However, whether IRAEs are predictive of response to ICIs remains controversial, as prospective cohort studies are lacking to prove this concept.

Our team believes this patient with stage IV urothelial cancer achieved a complete response 10 months after discontinuation of avelumab, which we assumed to be a late response to immunotherapy. We do not believe that it was a response to enfortumab vedotin because the patient only had one administration. He did not complete even 1 cycle of treatment and this is not a pattern of response expected with antibody drug conjugates. Besides, even before starting enfortumab vedotin, the patient had already presented a reduction in pulmonary micronodules in the baseline CT, as described earlier. 

In this case, we observe that during the window of time for immunotherapy to initiate its effects, the patient presented with new lung metastasis due to the cessation of chemotherapy, indicating a highly active and aggressive disease. Probably, the micrometastatic phenomenon was already ongoing after the patient stopped chemotherapy and ICIs did not have enough time to take action on the disease course. Also, he only had two administrations of avelumab and had to stop. Precisely, eight months after avelumab suspension, we can see a first response to immunotherapy, and 10 months later, a complete response, which is a little later than predicted from clinical trials (two to six months). Immunotherapy takes time to take action because immune activation is necessary and some cell-to-cell interactions need to be enhanced for it to be effective. We also know from the literature that response to immunotherapy, when obtained, will last over time due to the immunological memory. That also happened in this case with a maintained complete response for five months already. This is not expected with antibody-drug conjugates and even less with only one administration.

In conclusion, this type of response and window of time for treatment response is more compatible with a late response to immunotherapy, which we believe to be the case with this patient. This is an unexpected outcome in urothelial cancer according to the available data. We found no reports of any clinical case like this in the literature, and the final data from the Javelin 100 bladder trial reported a complete response in only 7.1% of its patients [15].

## Conclusions

This case reports a very rare immune-related adverse event and an uncommon complete response in a tumor as aggressive as urothelial carcinoma. The case reinforces how a multidisciplinary approach is crucial for rapid recognition and action to avoid serious consequences of diabetic ketoacidosis secondary to ICI. Further studies are required to identify which patients are at higher risk for serious immune adverse events and to explain why only some of these patients present better responses to ICI. More research is needed to clarify which immune interactions and mechanisms underlie unique late responses to ICIs, such as the one reported in this clinical case.
